# Cognitive Impairment and Pain Discrepancies in ADRD: Evaluating Pain Responses to tDCS Intervention

**DOI:** 10.1093/geroni/igaf122.2217

**Published:** 2025-12-31

**Authors:** Juyoung Park, Chiyoung Lee, Jason Hoang, Hyochol Ahn

**Affiliations:** University of Arizona, Tucson, Arizona, United States; University of Arizona, Tucson, Arizona, United States; University of Arizona, Tucson, Arizona, United States; University of Arizona, Tucson, Arizona, United States

## Abstract

Assessing pain in individuals with Alzheimer’s disease and related dementias (ADRD) is challenging due to cognitive impairments that reduce the reliability of self-reported pain ratings, such as the Numerical Rating Scale (NRS). While caregiver-observed assessments, such as the Mobilization-Observation-Behavior-Intensity-Dementia-2 (MOBID-2), provide options, discrepancies between self-reported and observer-rated pain across cognitive impairment levels remain understudied. This study examines how cognitive function, stratified by the Montreal Cognitive Assessment (MoCA-30), influences pain reporting and response to transcranial direct current stimulation (tDCS), a nonpharmacological pain intervention. In this double-blind, randomized, sham-controlled trial, 40 older adults with ADRD and chronic pain (tDCS: n = 20, Sham: n = 20) received home-based tDCS (2mA, 20 minutes/session) or sham stimulation for five consecutive days. Pain intensity was assessed at baseline, post-intervention, and 1-, 2-, and 3-month follow-ups using NRS and MOBID-2. Participants were categorized into mild (18–25) and moderate (10–17) cognitive impairment groups. The sample included 29 females, 36 White participants, and 23 married individuals. Results showed that cognitive impairment significantly moderated discrepancies between NRS and MOBID-2 (F(4,35) = 3.364, p = .020, η² = .278). A significant Time × MoCA Severity interaction (p = .012) indicated that participants with lower MoCA scores demonstrated greater discrepancies in pain ratings. MOBID-2 consistently detected pain reduction following active tDCS, whereas NRS was less sensitive to these changes, particularly in those with greater cognitive impairment. These findings highlight the importance of cognitive-adaptive pain assessments and reinforce tDCS as a promising nonpharmacological intervention for pain management in ADRD.

